# Insulin increases expression of cannabinoid receptor 1 by suppressing lysosomal degradation via ERK signaling pathway

**DOI:** 10.7150/ijms.126308

**Published:** 2026-05-01

**Authors:** Shui-Yu Liu, Ching-Heng Ting, Guey-Shyang Hwang, Chien-Wei Chen, Luen-Kui Chen, Pin-Hsuan Ho, Pin-Hsuan Lee, Guan-Lin Wu, Wei-Hsian Yin, Liang-Yi Wu, Kok-Min Seow, Heng-Fu Lin, Chi-Chang Juan

**Affiliations:** 1 Institute of Physiology, College of Medicine, National Yang Ming Chiao Tung University, Taipei 112304, Taiwan.; 2 Department of Pathology, MacKay Memorial Hospital, Taipei 104217, Taiwan.; 3 Department of Medicine, MacKay Medical College, New Taipei City 252005, Taiwan.; 4 Department of Nursing, Chang Gung University of Science and Technology, Taoyuan 333324, Taiwan.; 5 Department of Physical Education, Health, and Recreation, Teachers College, National Chiayi University, Chiayi 621302, Taiwan.; 6 Division of Cardiology, Cheng-Hsin General Hospital, Taipei 112401, Taiwan.; 7 Heart Center, Cheng-Hsin General Hospital, Taipei 112401, Taiwan.; 8 Department of Bioscience Technology, College of Science, Chung-Yuan Christian University, Chung Li 320314, Taiwan.; 9 Department of Obstetrics and Gynecology, Shin-Kong Wu Ho-Su Memorial Hospital, Taipei 111045, Taiwan.; 10 Department of Obstetrics and Gynecology, College of Medicine, National Yang Ming Chiao Tung University, Taipei 112304, Taiwan.; 11 Division of Trauma, Department of Surgery, Far-Eastern Memorial Hospital, New Taipei City 220216, Taiwan.; 12 Graduate Institute of Medicine, Yuan Ze University, Taoyuan 320315, Taiwan.; 13 Department of Medical Research, Taipei Veterans General Hospital, Taipei 112201, Taiwan

**Keywords:** cannabinoid receptor 1, ERK, hepatocytes, insulin, lysosomal pathway

## Abstract

Overactivation of the endocannabinoid (EC) system is an important pathogenic factor in the development of obesity. The inactivation of the EC system through administration of cannabinoid receptor 1 (CB1R) antagonist or CB1R knockout can ameliorate obesity-associated metabolic disorders such as insulin resistance and compensatory hyperinsulinemia and hepatic steatosis. However, the specific mechanisms by which hyperinsulinemia drives the progression of metabolic dysfunction-associated steatotic liver disease (MASLD) through CB1R remains poorly understood. The purpose of this study was to investigate the mechanism by which insulin regulates CB1R expression in hepatocytes. We studied the time and dose effects of insulin on CB1R expression in AML12 hepatocytes. Using specific inhibitors that act on insulin signaling pathways, we clarified the involvement of downstream signaling molecules in insulin-regulated CB1R expression. We also studied the effects of insulin on CB1R mRNA expression using real-time RT-PCR assays. In an *in vivo* study, we administered a hyperinsulinemic-euglycemic infusion to rats for 8 h, then measured CB1R expression in their hepatocytes. The insulin significantly increased the CB1R expression in AML12 and primary hepatocytes, and also in the rats' livers. Pretreatment with ERK inhibitors abolished this insulin-stimulated CB1R expression. Insulin suppressed the protein degradation of CB1R by inhibiting the lysosomal degradation pathway. Additionally, the activation of CB1R with selective agonists enhanced its lipogenic effects on insulin-pretreated hepatocytes. In conclusion, insulin increases hepatic CB1R expression by activating the ERK-dependent pathway and suppressing lysosomal degradation. This insulin-mediated CB1R stabilization provides a novel mechanistic link between hyperinsulinemia and the exacerbation of MASLD/MASH, suggesting that the hepatic insulin-ERK-CB1R axis may be a potential therapeutic target for metabolic liver diseases.

## Introduction

Insulin resistance is widely regarded as the main pathogenic factor in the development of metabolic syndrome and diabetes mellitus. Insulin is an endocrine hormone that acts on metabolic tissues (such as the liver), adipose tissue, and skeletal muscle; it also regulates glucose and lipid metabolism. The liver plays an important role in the regulation of glucose and lipid homeostasis in the body. For instance, impaired insulin function enhances hepatic glucose production and lipid accumulation 1, 2. Compensatory hyperinsulinemia is a noteworthy phenomenon driven by an insulin-resistant state. Furthermore, pathological hyperinsulinemia may suppress hepatic triglyceride (TG) output and cause hepatic steatosis 3.

The endocannabinoid (EC) system is involved in the pathogenesis of obesity, metabolic syndrome, and type 2 diabetes 4-7. EC system promotes lipogenesis 8 and regulate glucose homeostasis through the modulation of cannabinoid receptor activities 9, 10. The overactivation of cannabinoid receptor 1 (CB1R) likely leads to the deterioration of metabolic functions 7, 11, 12. CB1R-selective antagonists have been shown to reduce body weight and improve metabolic profiles in both animal and clinical trials 13-17. CB1R is widely expressed in various tissues, including the nervous system, gastrointestinal tract, adipose tissue, skeletal muscle, and pancreatic tissue 18, 19. It is also expressed in hepatocytes 20. Direct stimulation of hepatic CB1R enhances lipogenesis and lipid accumulation in hepatocytes 21. Hepatic CB1R is upregulated in human nonalcoholic steatohepatitis (NASH) and murine diet-induced-obesity models 22, 23. Furthermore, selectively knocking out CB1R in hepatocytes ameliorates diet-induced insulin resistance, hyperlipidemia, and hepatic steatosis 24. This indicates that hepatic CB1R is directly involved in systemic metabolic regulation.

Clinical and animal studies have suggested that insulin is strongly correlated with EC system activity. Both insulin resistance and compensatory hyperinsulinemia are hallmarks of metabolic syndrome. A hyperactive EC system has also been observed in patients with these symptoms 4. An epidemiological study revealed that serum EC levels are positively correlated with body mass index, serum insulin levels, and cardiovascular risks 25, 26. The expression of cannabinoid receptors, especially CB1R, in adipose tissue is also positively correlated with insulin resistance 26. However, the direct effects of insulin on EC system activity require further investigation. In this study, we found that insulin increased CB1R expression in cultured hepatocytes. We demonstrated the function of insulin in the regulation of hepatic EC system activity. We also revealed the possible pathogenesis of obesity-related EC system dysregulation.

## Materials and Methods

### Experimental design

Immortalized murine AML12 hepatocytes were incubated at various insulin concentrations (0, 10^-10^, 10^-9^, 10^-8^, and 10^-7^ M) for various durations (0, 4, 8, 12, 16, and 24 h) to investigate the effects of insulin on hepatic CB1R expression in DMEM/F12 medium. CB1R protein density was determined via western blot analysis. Primary rat hepatocytes were also used to evaluate the effects of insulin on CB1R density. To elucidate the underlying mechanism of insulin action, AML12 hepatocytes were pre-incubated for 1 h in the presence or absence of a PI3K inhibitor (LY294002) or ERK inhibitor (PD98059). The hepatocytes were then treated with 100 nM insulin for 24 h in the continuous presence or absence of the inhibitors, and CB1R protein density was determined via western blot.

To further confirm the involvement of ERK in insulin action, we used phorbol 12-myristate 13-acetate (PMA) to stimulate ERK phosphorylation and CB1R protein density was determined. Also, PD98059 was used to clarify the role of ERK in PMA-regulated CB1R density. To exclude the involvement of PKC, AML12 hepatocytes were pre-incubated for 1 h in the presence or absence of a PKC inhibitor (Ro-318220), the hepatocytes were then treated with or without 100 nM insulin for 24 h in the continuous presence or absence of the inhibitor, and CB1R protein density was determined via western blot. To further evaluate the effect of insulin on CB1R mRNA expression, AML12 hepatocytes were incubated with or without insulin (100 nM) for 24 h, and the CB1R mRNA expression was determined via real-time reverse transcriptase PCR.

To investigate the regulation of translation in the insulin-mediated upregulation of CB1R, AML12 hepatocytes were treated with 100 nM insulin in the presence or absence of 5 μg/mL cycloheximide (CHX) for various amounts of time (0-48 h). The treated cells were collected and CB1R protein density was analyzed via western blot. To identify the degradation pathway mediating the insulin-induced upregulation of CB1R, AML12 hepatocytes were pre-incubated for 1 h in the presence or absence of various inhibitors acting on different protein degradation pathways. The AML12 hepatocytes were then incubated with 100 nM insulin and 5 μg/mL CHX in the continued presence or absence of the inhibitors for an additional 24 h, and CB1R protein density was determined via western blot. The inhibitors used were the translation inhibitor CHX (5 μg/mL), the proteasome inhibitor MG132 (1 μM), and the lysosome inhibitor chloroquine (50 μM).

To investigate the involvement of ERK signaling in insulin-mediated CB1R degradation, the AML12 hepatocytes were pre-incubated with CHX (5 μg/mL) for 1 h in the presence or absence of PD98059. The hepatocytes were treated with 100 nM insulin for 24 h in the continuous presence or absence of the inhibitor and CB1R protein density was then measured via western blot. To examine the regulatory effect of insulin on hepatic CB1R density *in vivo*, we administered a co-infusion of insulin and glucose to conscious rats for 8 h to increase their circulating insulin levels. Saline-infused rats served as controls. The rats were sacrificed after saline infusion; their livers were removed and their hepatic CB1R density was determined via western blot.

To examine the physiological consequences of insulin-stimulated CB1R density in hepatocytes, we analyzed the effect of pharmacological activation of CB1R on hepatic TG content using arachidonyl-2-chloroethylamide (ACEA). We also used oleic acid (OA) to stimulate TG accumulation. After 24 h of pre-incubation with or without 100 nM insulin in the presence or absence of PD98059, the cells were washed thrice with phosphate-buffered saline (PBS) and incubated in a medium containing 200 μM OA in the presence or absence of the CB1R-selective agonist ACEA. The intracellular TG content was observed and quantified via oil red O staining and TG assay kits.

### Materials

The cannabinoid-receptor agonist ACEA was purchased from TOCRIS Bioscience (Ellisville, MO, USA). The cannabinoid receptor antagonists rimonabant and AM630 were obtained from Cayman Chemical (Ann Arbor, MI, USA). The anti-CB1R antibody was from Cell Signaling Technology (catalog no. #93815, RRID: D5N5C; Danvers, MA, USA). Table 1 lists the other antibodies used, including their sources, host species, and dilutions. All other reagents used were purchased from Sigma-Aldrich (St. Louis, MO, USA) unless otherwise stated.

### Cell culture

The mouse immortalized AML12 (ATCC® CRL-2254™, RRID: CVCL_0140) hepatocyte cell line was cultured in DMEM/F12 (GibcoTM, Cat. No. 12500062; glucose: 17.5 mM) containing 10% (v/v) fetal bovine serum, 10 μg/mL insulin, 6.7 ng/mL sodium selenite, 5.5 μg/mL transferrin, and 40 ng/mL dexamethasone. Before the experiment was conducted, the cells were incubated in serum-free DMEM/F12 without supplements for 16 h to achieve quiescence. The AML12 hepatocytes were treated with various doses of insulin for various durations to examine the effects of insulin. To investigate whether certain pathways mediate insulin-stimulated CB1R densities, we treated the cells with inhibitors for 1 h before the insulin (100 nM) was administered. Twenty-four hours after the insulin was administered, the cells were collected and subjected to analysis.

### Animals

Eight-week-old male Sprague-Dawley (SD) rats weighing 250-300 g were purchased from the Laboratory Animal Center of National Yang Ming Chiao Tung University. Four rats were housed in each cage at 20-22 °C in a light-controlled room kept on a 12 h/12 h light/dark cycle (lights were turned on at 08:00). The rats were fed with a regular chow diet and water *ad libitum*. All animal experiments and laboratory procedures were conducted in accordance with the guidelines on animal care including the ARRIVE guidelines and use as approved by the Institutional Animal Care and Use Committee of National Yang Ming Chiao Tung University (IACUC approval number: 1140323). Rats were anesthetized with an intraperitoneal injection of sodium pentobarbital (3 mg/100 g body weight) prior to surgical procedures. At the end of the experiments, animals were euthanized via anesthetic overdose to ensure minimal pain and distress, in compliance with ARRIVE guidelines.

### Isolation and culture of primary rat hepatocytes

After the rats were anesthetized (sodium pentobarbital, 3 mg/100 g BW, i.p.), a laparotomy and portal vein catheterization were performed with a polyethylene tube (PE-50). The rat liver was perfused with a 37 °C calcium-free Hank's balanced salt solution (HBSS) and then with 37 °C, 0.1% collagenase IV in HBSS. The liver was minced and the hepatocytes were centrifuged and resuspended in DMEM/F12 containing 10% (v/v) fetal bovine serum and supplement.

### Insulin/glucose co-infusion to maintain a hyperinsulinemic-euglycemic state

The day before the experiment, the rats were anesthetized (sodium pentobarbital, 3 mg/100 g BW, i.p.) and the right jugular and left femoral veins were cannulated to enable blood collection and infusion administration, respectively. In the morning, a reference blood sample was collected, after which a mixed solution of 0.27 mU of insulin and 0.8 mg of glucose/100 g body weight was infused per minute for 8 h at a rate of 1 mL/h. Saline-infused rats were used as a control group. After the 8 h infusion, blood samples were collected using an intravenous catheter and placed in 1.5 mL heparin-coated polyethylene microcentrifuge tubes on ice for glucose and insulin measurements. All rats were then sacrificed and their livers were collected to measure CB1R density. Plasma was separated via centrifugation and stored at -20 °C until the assay was performed. The plasma insulin concentration was determined using a commercial rat insulin ELISA kit (Mercodia AB, Uppsala, Sweden). Plasma glucose was measured using a glucose analyzer (Model 23A, Yellow Springs Instruments, Yellow Springs, OH, USA).

### Western blots

Cell lysate was collected using a lysis buffer (1% Triton X-100, 150 mM NaCl, 100 mM Tris, 1 mM EDTA, 1 mM EGTA, 0.2 mM sodium ortho-vanadate, 0.2 mM PMSF, 0.5% NP-40) and then homogenized. The solubilized protein was harvested; the protein concentration was determined and equalized using the Bradford method (Bio-Rad Protein Assay). The samples were resolved in 10% SDS-PAGE. The contents of the gel were transferred to a polyvinylidene difluoride membrane. The membrane was pre-blotted in skim milk buffer and then immunoblotted with anti-CB1R (Cell Signaling Technology, Catalogue No. #93815, RRID: D5N5C), p-ERK, p-Akt, and GAPDH primary antibodies. Subsequently, the blots were stripped and re-probed with the total forms of ERK and Akt. Horseradish peroxidase-conjugated secondary antibodies were then used in conjunction with a chemiluminescent reagent.

### RNA extraction and real-time RT-PCR

The AML12 hepatocytes were lysed and total RNA was extracted using a Tri Reagent kit (Applied Biosystems, Waltham, MA, USA). The RNA concentration was determined based on UV light absorption at 260 nm and the integrity of the extracted total RNA was examined via 1% agarose gel electrophoresis. The A260/280 ratios were between [1.8-2.0], confirming high purity. The RNA samples were reverse-transcribed with random primers and reverse transcriptase (RT; Applied Biosystems) to obtain the cDNA product. Real-time PCR was performed using a StepOnePlus Real-Time PCR System (Applied Biosystems) via the comparative *C*t quantification method. *Taq*Man gene expression assays (Applied Biosystems) with specific primers (assay ID for mouse CB1R: Mm01212171_s1; for GAPDH: Mn99999915_g1), *Taq*Man MGB probe (FAM dye-labelled), *Taq*Man Fast Universal PCR Master Mix, and 100 ng of cDNA were used to detect and quantify the mRNA levels of CB1R in the AML12 hepatocytes. The reaction conditions were as follows: 95 °C for 10 min followed by 40 cycles of 95 °C for 15 s and 60 °C for 1 min. The mRNA of GAPDH was amplified and used as an internal control.

### Oil red O staining

Oil red O powder was dissolved in 2-propanol and filtered to obtain the staining solution. The cells were washed with PBS and fixed with 4% paraformaldehyde. Fixed cells were stained with the oil red O solution for 30 min. Intracellular oil red O was observed under a light microscope. To quantify the oil red O staining results, we washed the stained cells with distilled water and added 1 mL of isopropanol for 10 min to extract the intracellular oil red O. The optical density of the extract solution at 510 nm was determined using a spectrophotometer. The ACEA treatment as the positive control for lipid accumulation.

### Triglyceride content determination

Triglyceride content was measured using the Triglyceride FS reagent (triglyceride enzymatic kit, Diagnostic Systems GmbH, Holzheim, Germany). An aliquot of 5 μl cell extract was added to 250 μl reagent and incubated at room temperature for 20 minutes. Absorbance was measured at 550 nm using a spectrophotometer, and triglyceride concentrations were calculated from a standard curve generated using the Multi-system calibrator (Diagnostic Systems GmbH).

### Blood sampling and biochemical analysis

Blood samples were collected using an intravenous catheter and placed in 1.5 mL heparin-coated polyethylene microcentrifuge tubes on ice for glucose and insulin measurements. Plasma was separated via centrifugation and stored at -20 °C until the assay was performed. Plasma glucose concentration was determined using a glucose analyzer (Model 23A; Yellow Springs Instruments). Plasma insulin concentration was determined using a commercial ELISA kit (Mercodia AB, Uppsala, Sweden).

### Statistical analysis

Cell studies were repeated at least thrice. The sample size was six rats per group. Statistical analysis is based on biological replicates. Results were expressed as mean ± SEM. Statistical significance was assessed using one-way analysis of variance (ANOVA) followed by the Tukey's post hoc test or Student's *t*-tests. Statistical significance was assumed at *p* < 0.05.

## Results

### Insulin stimulates CB1R protein density in hepatocytes

**Figure 1:**
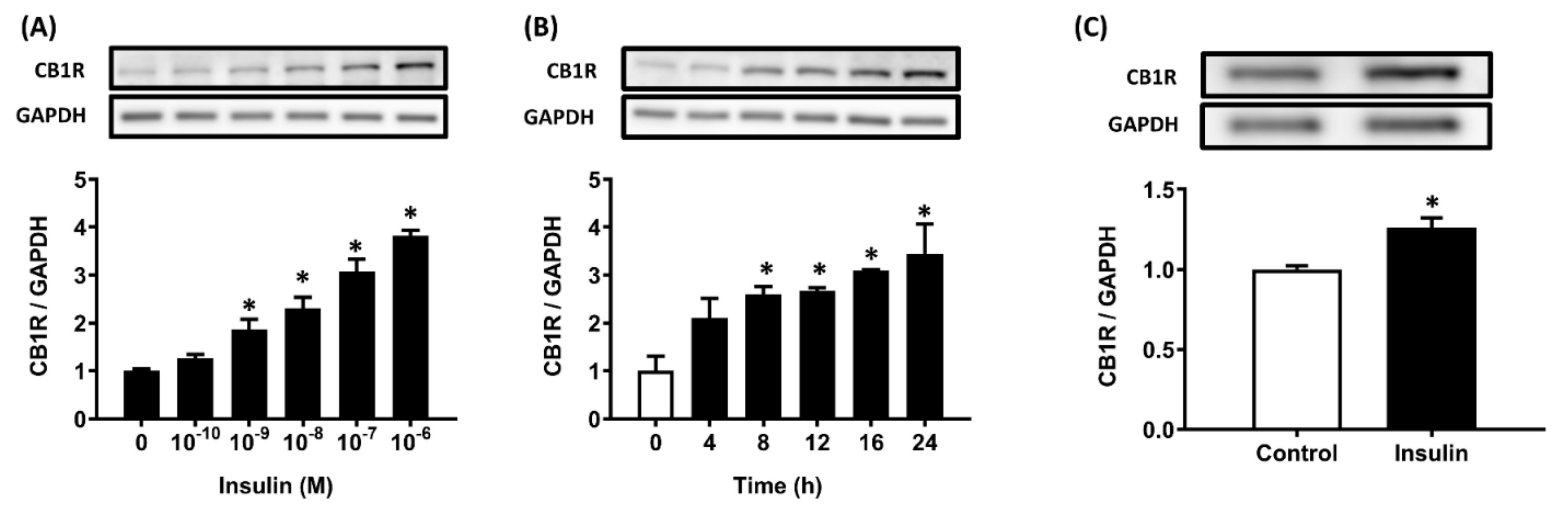
Insulin-stimulated CB1R expression in AML12 and primary hepatocytes. Hepatocytes were treated with the indicated concentration of insulin for 24 h (A) or 10^-7^ M insulin for the indicated time (B) and CB1R protein levels were then measured via western blot. The effect of insulin on CB1R expression was also examined in primary hepatocytes after 24 h of insulin (10^-7^ M) incubation (C). The results are expressed as fold change (mean ± SEM) from four biologically independent experiments. GAPDH served as a loading control. * *p* < .05 compared with time zero or vehicle controls.

The immortalized AML12 hepatocytes were treated with 100 nM insulin for various durations (0-24 h) or various insulin concentrations (0-100 nM) for 24 h in DMEM/F12 medium (glucose: 17.5 mM). A significant increase in the CB1R protein level was first seen after 8 h of insulin incubation (*p* < .05), and levels increased until 24 h (Figure 1A). AML12 hepatocytes were then incubated for 24 h with various concentrations of insulin (0-10^-7^ M). Incubation with 10^-9^ M insulin resulted in a significant increase in CB1R protein levels compared to incubation without insulin (*p* < .05; Figure 1B). Thus, insulin increased CB1R protein densities in a time- and dose-dependent manner (Figure 1A, B). We further examined the stimulatory effects of insulin on CB1R densities in primary rat hepatocytes and found that it had the same effect there (Figure 1C).

### Insulin increases hepatic CB1R density via the ERK-mediated pathway

**Figure 2:**
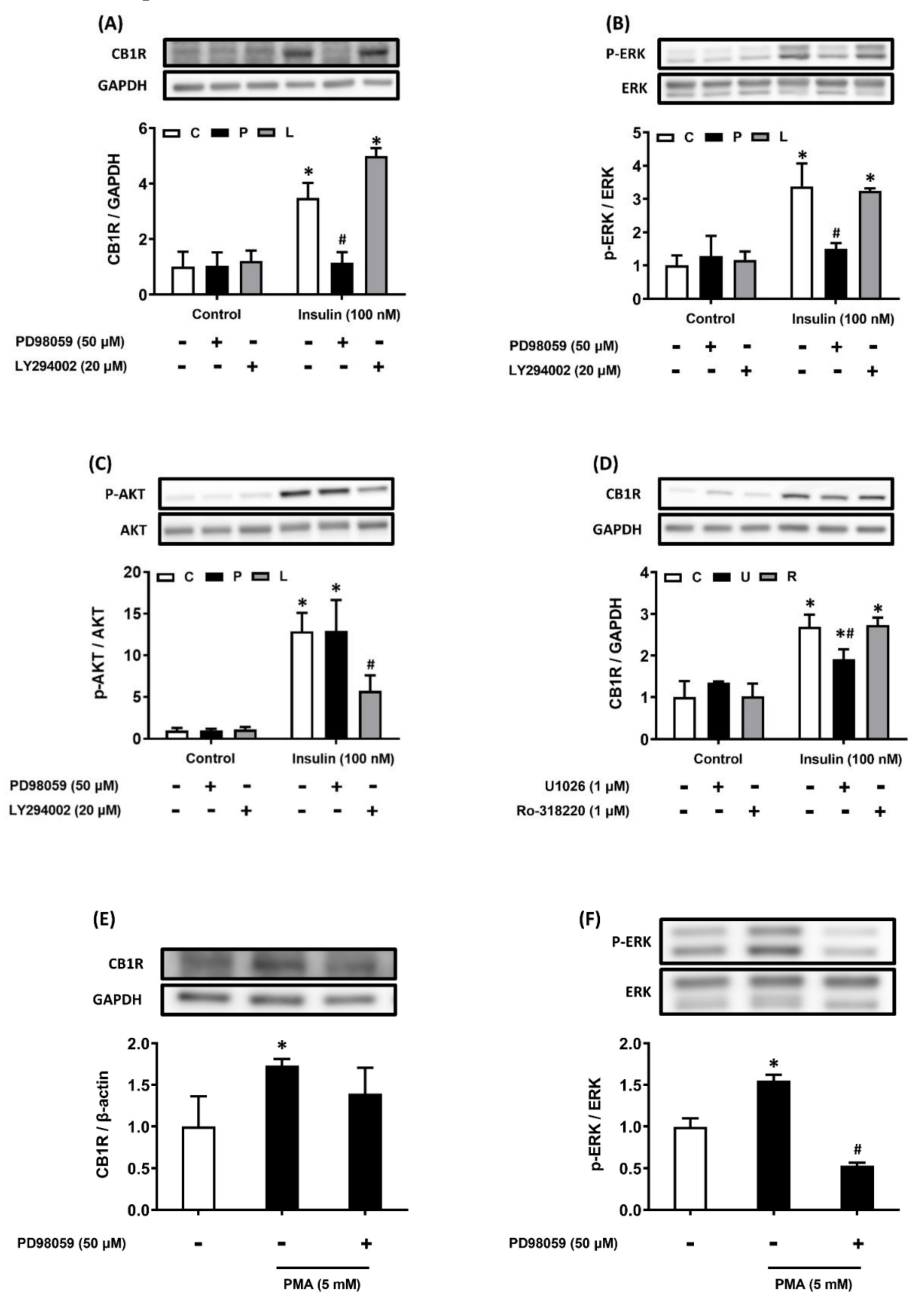
Insulin stimulates CB1R expression via the ERK-mediated pathway. AML12 hepatocytes were preincubated for 1 h in the presence or absence of the ERK inhibitor PD98059 (P, 50 μM) or the PI3K inhibitor LY294002 (L, 20 μM) and then incubated for another 15 min or 24 h in the presence or absence of 100 nM insulin in the continued presence or absence of the inhibitor (A-C). To further confirm the function of ERK in insulin-regulated CB1R expression, AML12 hepatocytes were preincubated for 1 h in the presence or absence of another ERK inhibitor, U0126 (1 μM), and then incubated for another 24 h in the presence or absence of 100 nM insulin in the continued presence or absence of the inhibitor (D). AML12 hepatocytes were preincubated for 1 h in the presence or absence of the PKC inhibitor Ro-318220 (1 μM) and then incubated for another 24 h in the presence or absence of insulin (100 nM) in the continued presence or absence of Ro-318220 (D). AML12 hepatocytes were preincubated for 1 h in the presence or absence of the ERK inhibitor PD98059 (50 μM) and then incubated for another 15 min (to detect ERK phosphorylation) or 24 h (to detect CB1R) in the presence or absence of the PKC activator PMA (5 mM) and in the continued presence or absence of PD98059 (E and F). Density levels of ERK, p-ERK, Akt, p-Akt and CB1R were measured via western blot. GAPDH, ERK, Akt served as a loading control. The blot shown is representative of the results of four independent experiments. The results are expressed as fold change (mean ± SEM) from four biologically independent experiments. * *p* < .05 compared with the vehicle control; ^#^ *p* < .05 compared with insulin alone.

After AML12 hepatocytes had been pretreated with the PI3K and ERK pathway inhibitors, they were treated with 100 nM insulin. Insulin-induced CB1R density was diminished after ERK pathway inhibition, but not after PI3K pathway inhibition (Figure 2A-C). We used U0126, another ERK inhibitor, to confirm these results: pretreatment with U0126 also suppressed insulin-induced CB1R density in the hepatocytes (Figure 2D). Considering that PMA treatment can also activate cellular protein kinase C (PKC), we investigated whether PKC activation was involved in the regulation of insulin-stimulated CB1R density in hepatocytes. However, we found it was not affected by pretreatment with the PKC inhibitor Ro-318220 (Figure 2D). We used PMA to activate the endogenous ERK pathway and further explore the role of the ERK pathway in insulin-stimulated CB1R density. This treatment stimulated ERK phosphorylation, which can be inhibited by PD98059, an ERK inhibitor. After 24 h of treatment with PMA, CB1R density increased, but this increase was blocked by PD98059 (Figure 2E, F). This suggested that ERK signaling is important for the regulation of CB1R density. Collectively, these results showed that the insulin-stimulated upregulation of hepatic CB1R density is dependent on the ERK-mediated pathway.

### Insulin does not increase hepatic CB1R expression at the transcriptional level

**Figure 3:**
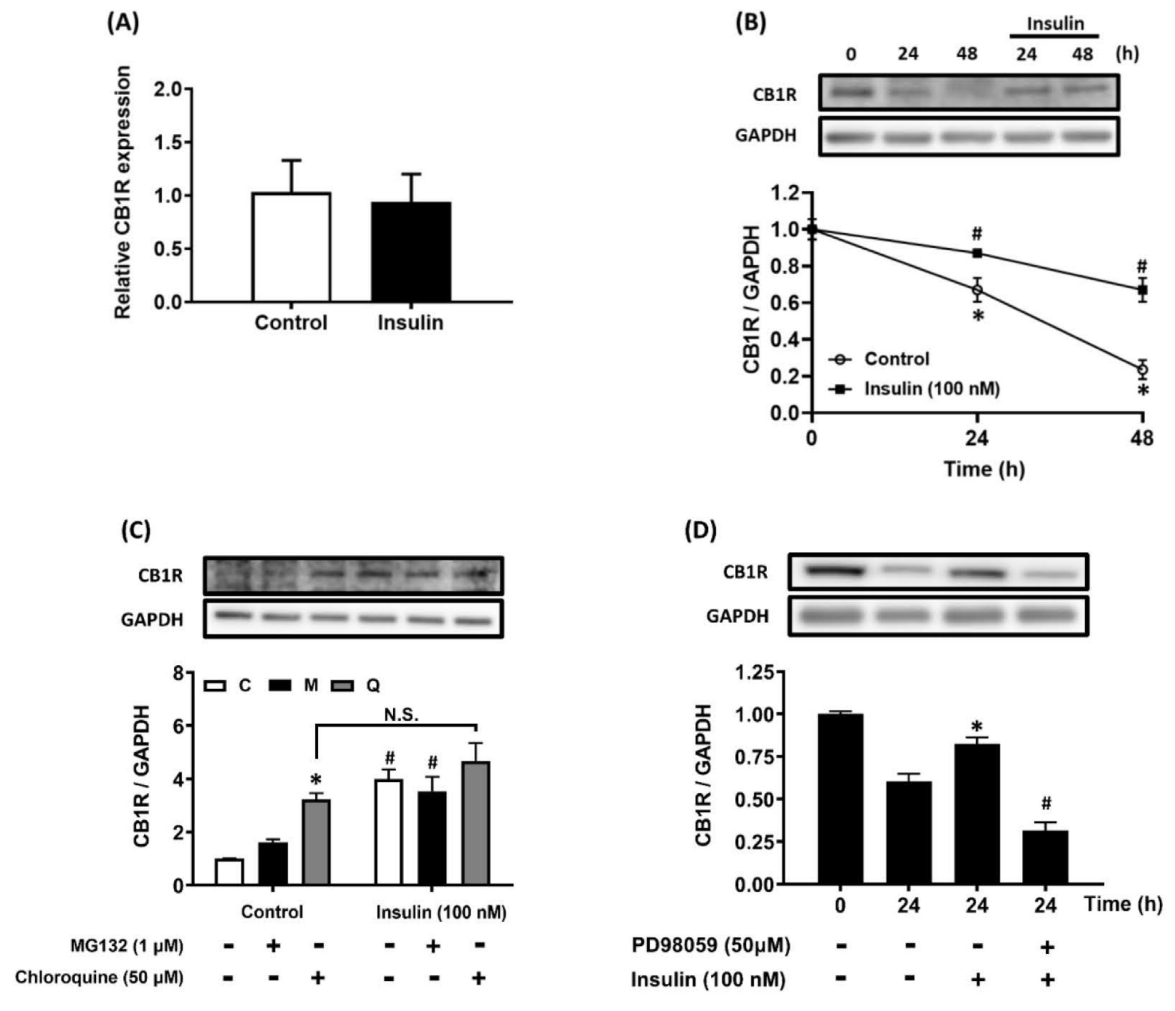
CB1R mRNA expression is not regulated by insulin and insulin prevents CB1R protein degradation by inhibiting the lysosomal degradation pathway. (A) AML12 hepatocytes were incubated with insulin (100 nM) for 24 h and CB1R mRNA expression was measured via real-time RT-PCR. (B) AML12 hepatocytes were preincubated with the protein-synthesis inhibitor cycloheximide (5 μg/ml) for 1 h and then incubated for another 24 h or 48 h in the presence or absence of 100 nM insulin in the continued presence or absence of cycloheximide. (C) To further clarify the effect of insulin-increased CB1R density on protein-degradation pathways, AML12 hepatocytes were preincubated with the protein-synthesis inhibitor cycloheximide (5 μg/ml) for 1 h. The hepatocytes were then preincubated for another 1 h in the presence or absence of the proteasomal degradation inhibitor MG132 (M, 1 μM) and the lysosomal degradation inhibitor chloroquine (Q, 50 μM). Finally, they were incubated for another 24 h in the presence or absence of 100 nM insulin in the continued presence or absence of the inhibitors. (D) AML12 hepatocytes were preincubated with cycloheximide (5 μg/ml) in the presence or absence of the ERK inhibitor PD98059 (50 μM) for 1 h and then incubated with 100 nM insulin for another 24 h in the continued presence or absence of the inhibitors. The expression of CB1R was measured via western blot. GAPDH served as a loading control. The results are expressed as fold change (mean ± SEM) from four biologically independent experiments. * *p* < .05 compared with the vehicle control; ^#^ *p* < .05 compared with insulin alone.

The AML12 hepatocytes were treated with 100 nM insulin for 24 h to analyze the effects of insulin on the expression of CB1R mRNA. The mRNA levels were analyzed using real-time RT-PCR. There was no statistically significant difference in CB1R mRNA expression between the insulin-treated and control groups. This suggests that insulin does not regulate the transcription of CB1R mRNA in hepatocytes (Figure 3A).

### The lysosomal degradation pathway is involved in insulin-stimulated CB1R density

The degradation rate of CB1R was measured after *de novo* protein synthesis was inhibited with cycloheximide. The degradation of CB1R in the presence of CHX and insulin was slower than that without insulin treatment over a period of 24-48 h (Figure 3B). Further analysis of the involvement of the proteasome- and lysosome-mediated degradation pathways in protein stability showed that insulin-stimulated CB1R density was significantly blocked by chloroquine, but not by MG132, in the presence of CHX (Figure 3C). This suggested that CB1R density is increased by insulin through the suppression of lysosome-mediated protein degradation. Furthermore, insulin-suppressed CB1R protein degradation was significantly inhibited by pretreatment with PD98059 (Figure 3D). Therefore, insulin-suppressed CB1R protein degradation is ERK-dependent and mediated by the lysosomal degradation pathway but not the proteasomal degradation pathway.

### Insulin/glucose co-infusion increases hepatic CB1R expression *in vivo*

**Figure 4:**
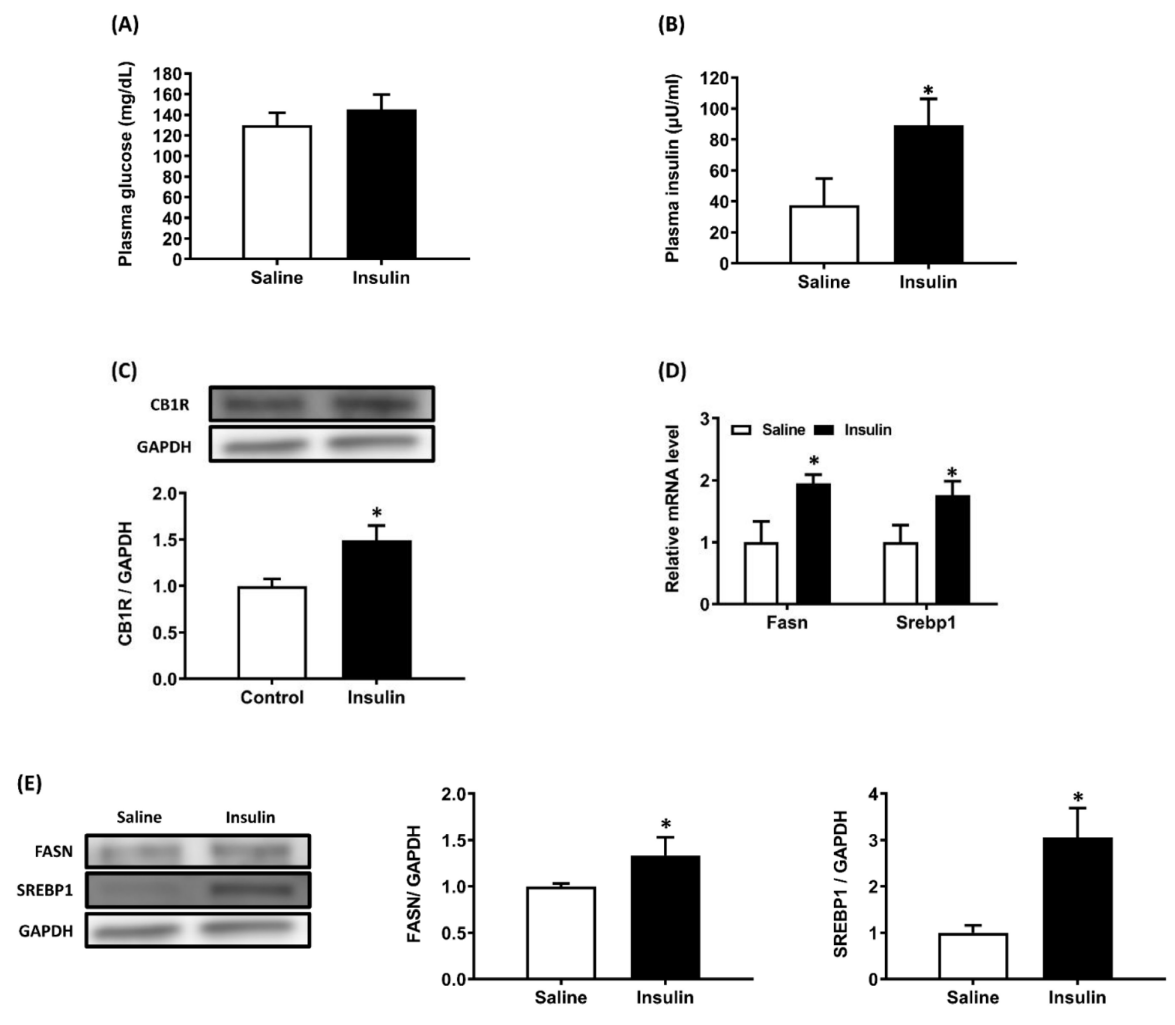
Increased hepatic CB1R expression in conscious rats after 8 h of hyperinsulinemic-euglycemic treatment. Conscious rats received an insulin/glucose co-infusion for 8 h to simulate an endogenous hyperinsulinemic-euglycemic state. Rats infused with saline alone were used as the control group. After 8 h of infusion, both groups of rats were sacrificed and their plasma insulin, glucose (A, B), hepatic CB1R density (C), and fatty acid synthase (Fasn) and Srebp1 mRNA expression (D) and protein density (E) were measured. GAPDH served as a loading control. The blot shown is representative of the results. The results are expressed as fold change (mean ± SEM) from ix rats per group. * *p* < .05 compared with the saline-infused group (A and B) or control chow diet group (C).

Compared with the saline-infused group, 8 h of insulin/glucose co-infusion caused a hyperinsulinemic-euglycemic state in conscious rats (Figure 4A, 4B). Hepatic CB1R protein density was higher after 8 h of insulin/glucose co-infusion than in saline-infused controls (Figure 4C). Then we examined the expression of lipogenic genes in liver from both groups of rats and results showed that mRNA and protein expression/density of fatty acid synthase (Fasn) and Srebp1 in liver from insulin/glucose-co-infused rats were significantly increased compared with that from saline-infused controls (Figure 4D, 4E).

### Insulin enhances cannabinoid-induced lipid accumulation via the ERK-mediated pathway

**Figure 5:**
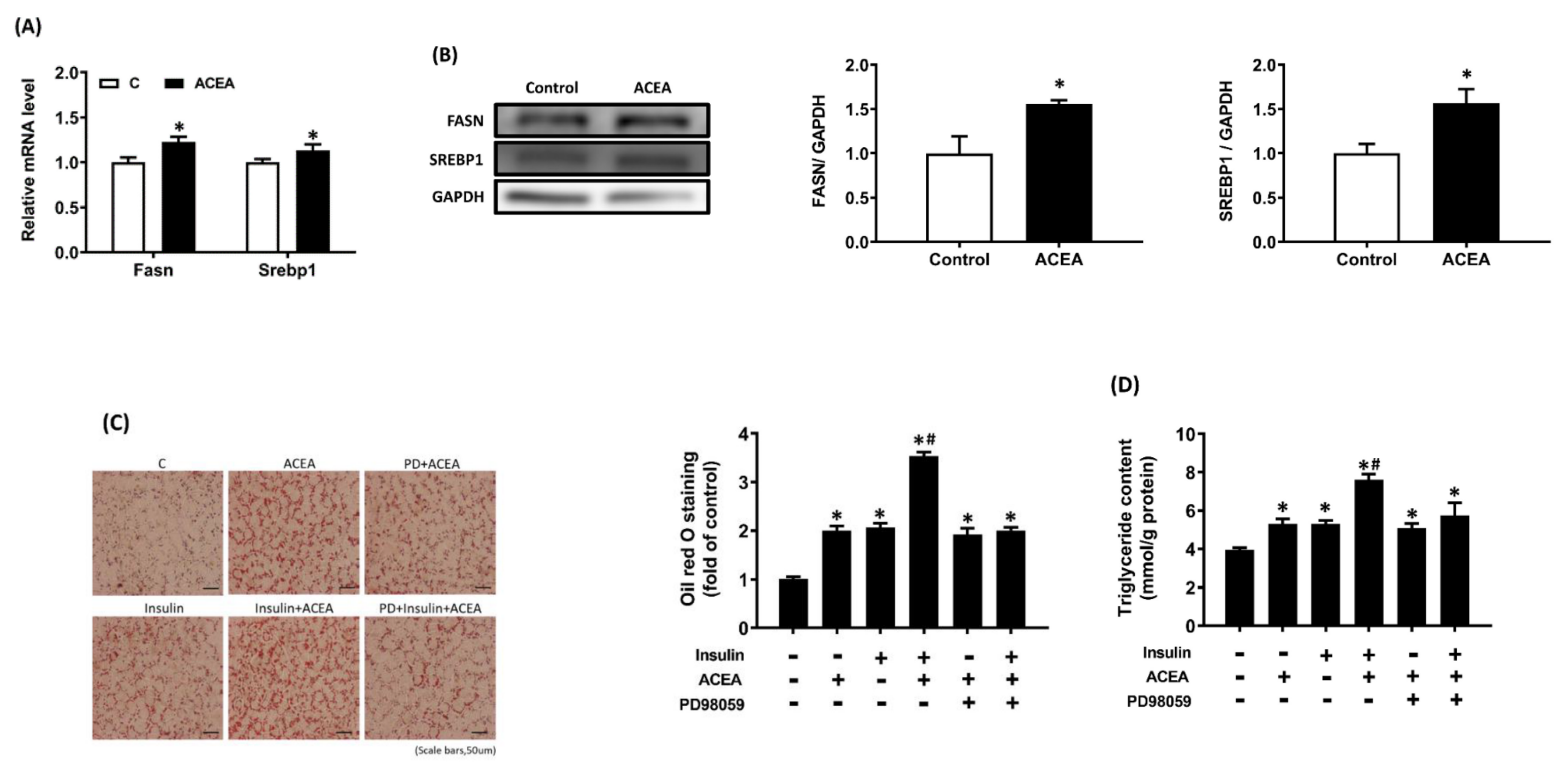
Insulin enhances intracellular lipid accumulation induced by the CB1R agonist arachidonyl-2-chloroethylamide (ACEA) in AML12 hepatocytes. AML12 hepatocytes were incubated in the presence of oleic acid (200 μM) with or without ACEA (1 μM) for 24 h, then Fasn and Srebp1 mRNA expression (A) and protein density (B) were measured. Then AML12 hepatocytes were preincubated for 1 h in the presence or absence of the ERK inhibitor PD98059, then treated with or without insulin (100 nM) in the continued presence or absence of PD98059 for 24 h, and finally incubated in the presence of oleic acid (200 μM) with or without ACEA (1 μM) for another 24 h. Intracellular triglyceride (TG) accumulation was evaluated via oil red O staining (C) and a TG assay kit (D). The blot shown is representative of the results (GAPDH served as a loading control). The oil red O-stained image shown is representative of the results. The results are expressed as fold change (mean ± SEM) from four biologically independent experiments. * *p* < .05 compared with the vehicle control; ^#^ *p* < .05 compared with insulin alone.

Based on the findings from insulin/glucose-co-infused rats, we further study the effect of CB1R activation on expression/density of Fasn and Srebp1 in AML 12 hepatocytes. Results showed that treatment with CB1R-selective agonist ACEA (1 μM) for 24 h significantly stimulated fatty acid synthase (Fasn) and Srebp1 gene expression and protein density (Figure 5A, 5B). Besides, after 24 h of insulin stimulation in the presence or absence of PD98059, the treated AML12 hepatocytes were washed with PBS and 200 μM oleic acid was added with or without the ACEA for 24 h. Results of oil red O staining showed that treatment with ACEA or insulin alone significantly increased lipid accumulation (Figure 5C). Insulin and ACEA cotreatment further increased lipid accumulation relative to the ACEA or insulin treatment alone (*p* < .05; Figure 5C). This additive effect was attenuated by pretreatment with PD98059 (Figure 5C). We observed similar effects on intracellular TG content (Figure 5D).

## Discussion

The present study was performed to explore the possible regulatory effect of insulin on CB1R expression in AML12 hepatocytes and to clarify the underlying signaling pathways. The effects of insulin on the regulation of the EC system are controversial. Di Marzo *et al*. demonstrated that insulin negatively regulates plasma EC levels in both obese and nonobese subjects 27. Insulin has also been reported to stimulate the expression of the EC-catabolizing enzyme fatty acid amide hydrolase (FAAH) but to have no effect on CB1R expression in human subcutaneous abdominal adipose tissue 28. Another human study found that circulating 2-arachidonoyl glycerol was positively correlated with fasting insulin levels and that CB1R mRNA expression in visceral adipose tissue was negatively correlated with fasting insulin levels 4. Our main finding was that insulin stimulated CB1R densities in hepatocytes in a time- and dose-dependent manner. Additionally, both ERK and Akt-dependent signaling have been suggested to be involved in the regulation of CB1R density. For example, EC system increased CB1R expression in striatal neurons in an Akt-dependent manner 29. Lim *et al*. found that the upregulation of CB1R in rats with chronic constriction sciatic nerve injury was ERK-dependent 30. Methamphetamine also increased CB1R mRNA expression in the striatum via the ERK-dependent pathway 31. In the current study, we demonstrated that insulin upregulated CB1R density via ERK activation but not via PI3K/Akt activation.

Potential downstream mediators of ERK signaling in the suppression of lysosomal CB1R degradation warrant further investigation. TFEB, a master regulator of lysosomal biogenesis that governs lysosome-related gene expression 32, is one candidate. Liu *et al*. 33 and Marchand *et al*. 34 demonstrated that ERK activation promotes TFEB phosphorylation and impairs its nuclear translocation. Another potential effector is TMEM55B, a phosphatidylinositol-4,5-bisphosphate 4-phosphatase that converts PtdIns-4,5-P_2_ to PtdIns-5-P and localizes to late endosomal/lysosomal membranes, where it contributes to lysosomal degradation following clathrin-mediated endocytosis 35. Takemasu *et al*. 36 further showed that TMEM55B is phosphorylated by ERK and regulates lysosomal clustering. Amino acid starvation induced perinuclear LAMP1 clustering in RAW264.7 macrophages, which was attenuated by TMEM55B knockdown or knockout, whereas TMEM55B overexpression enhanced clustering relative to wild type (Ref-5). Collectively, these findings indicate that ERK-dependent phosphorylation of TMEM55B modulates lysosomal dynamics. Together, these observations suggest that ERK-mediated regulation of TFEB nuclear translocation or TMEM55B expression may constitute a common downstream mechanism underlying the suppression of lysosomal CB1R degradation; however, further investigation is required to substantiate this hypothesis.

Further, we found that insulin did not stimulate an increase in CB1R mRNA expression. It may therefore regulate CB1R protein density via a post-translational mechanism. Some studies have reported a possible mechanism associated with the degradation of CBR proteins. For example, Chen *et al*. reported that the proteasome-mediated pathway may be involved in the regulation of CB2R protein degradation 37. Rozenfeld and Devi found that CB1R associates with the adaptor protein AP-3 and is trafficked to the lysosome 38. Most CB1Rs have been shown to be targeted by lysosomes for degradation 39. Furthermore, studies have also reported that insulin can regulate protein-degradation mechanisms. Mayer and Belsham demonstrated that insulin stimulated both proteasomal and lysosomal degradation to attenuate central insulin signaling in hypothalamic cell lines 40. Insulin also stimulated proteasomal and lysosomal degradation in mouse and human podocytes 41. However, other studies obtained contradictory findings indicating that insulin may act as a suppressor of both proteasomal and lysosomal degradation 42, 43. In the present study, we demonstrated that insulin suppressed the lysosomal degradation pathway to preserve the protein stability of CB1R in hepatocytes.

We further elucidated the physiological significance of insulin-upregulated CB1R density in hepatocytes by monitoring lipid accumulation. We found that treatment with insulin or ACEA alone significantly increased lipid accumulation (Figure 5). These results were consistent with previous findings that insulin facilitates fatty-acid uptake, promoting hepatic steatosis 7, and that CB1R agonists increase lipid accumulation in hepatocytes 21. After insulin treatment, the ACEA-stimulated lipid accumulation in hepatocytes was greater than in groups treated only with insulin or ACEA. Our interpretation of these results is that insulin-upregulated CB1R may cause an overactivation of CB1R and stimulate more fatty acid uptake and enhance lipid accumulation in hepatocytes. The current findings are consistent with those of Osei-Hyiaman *et al*. 24 and Zduniak *et al*. 44, where overexpressed and overactivated CB1R in hepatocytes contributed to the development of diet-induced steatosis.

**Figure 6:**
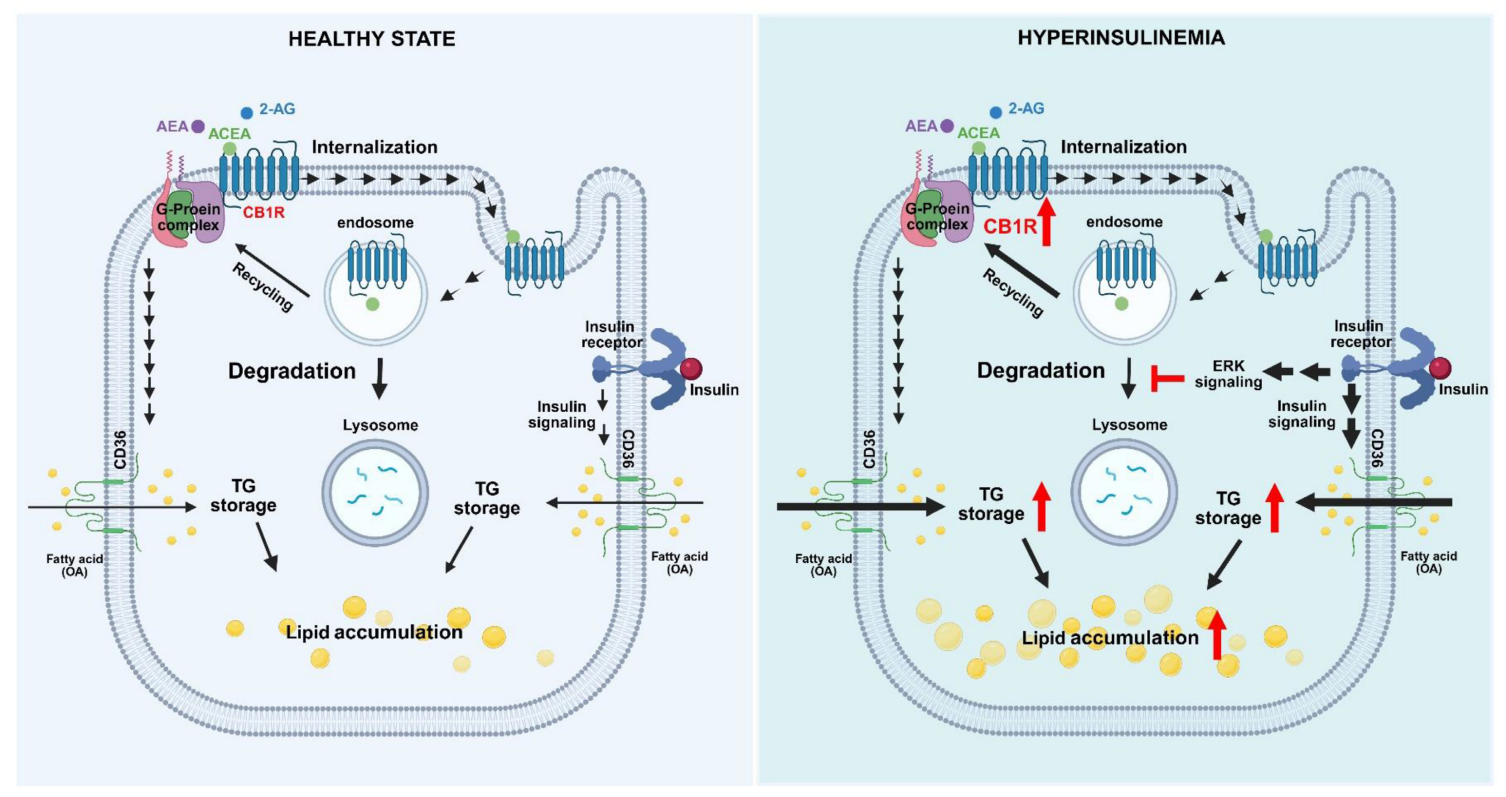
Hypothesized model of insulin-induced activation of the endocannabinoid system and its effects on lipid accumulation in hepatocytes. In states of hyperinsulinemia, insulin binds to an insulin receptor and evokes the serial activation of signaling molecules such as ERK, which subsequently suppresses the lysosomal degradation pathway and eventually leads to the upregulation of CB1R protein expression. Consequently, this further enhances EC-stimulated fatty-acid uptake and lipid accumulation.

In conclusion, we found that insulin increased the protein density of CB1R via an ERK-mediated mechanism in hepatocytes. The suppression by insulin of protein-degradation pathways was involved in insulin-induced CB1R overexpression. The increased CB1R expression could further trigger and enhance CB1R-agonist-induced lipid accumulation in hepatocytes (Figure 6). A recent study demonstrated that knockout of CB1R alleviates hepatic steatosis 45. In combination with our findings, these results suggest that CB1R is required for the development of hepatic steatosis. In addition to insulin-stimulated fatty acid uptake, insulin-resistance-compensatory hyperinsulinemia may increase hepatic CB1R expression in obesity. Furthermore, elevated EC system may stimulate increased fatty-acid uptake and then enhanced lipid accumulation in hepatocytes. This may contribute to the pathogenesis of fatty liver and other metabolic disorders. Thus, pharmacological inhibition of CB1R activation may represent a novel therapeutic approach for the treatment of fatty liver.

## Figures and Tables

**Table 1:** Primary and secondary antibodies used in western blot analysis

Primary Antibodies				
Target	Host	Dilution	Company	Catalog no.
CB1 Receptor (CNR1) (D5N5C)	Rabbit	1:1000	Cell Signaling Technology	93815
Phospho-p44/42 MAPK (ERK1/2) (Thr202/Tyr204)	Rabbit	1:1000	Cell Signaling Technology	9101
p44/42 MAPK (ERK1/2)	Rabbit	1:1000	Cell Signaling Technology	9102
Phospho-AKT (Ser473)	Rabbit	1:1000	Cell Signaling Technology	9271S
AKT (pan) (C67E7)	Rabbit	1:1000	Cell Signaling Technology	4691S
Fatty Acid Synthase (FASN) (C20G5)	Rabbit	1:1000	Cell Signaling Technology	3180S
Sterol regulatory element-binding protein 1 (SREBP-1)	Mouse	1:1000	Invitrogen (Thermo Fisher)	MA5-11685
GAPDH (14C10)	Rabbit	1:5000	Cell Signaling Technology	2118S
Secondary Antibodies				
Target	Host	Dilution	Company	Catalog no.
IgG-HRP, anti-mouse (H+L)	Goat	1:5000	SeraCare (KPL)	5220-0341
IgG-HRP, anti-rabbit (H+L)	Goat	1:5000	Millipore	AP132P

## Data Availability

All data and materials involved in this study can be found in this article. Additional information can be made available upon reasonable request.
